# Anatomical Study of the Relationship between the Riché-Cannieu Anastomosis and the Kaplan Cardinal Line

**DOI:** 10.1055/s-0044-1785512

**Published:** 2024-06-22

**Authors:** Edie Benedito Caetano, Luiz Angelo Vieira, Vinicius Santos Bueno, Giovanni Chammas Consorte, Luiz Felipe Ferreira Spalluto, Kilder Christofoli

**Affiliations:** 1 Departamento de Cirurgia, Faculdade de Ciências Médicas e da Saúde, Pontifícia Universidade Católica de São Paulo, Sorocaba, SP, Brasil; 2 Faculdade de Ciências Médicas e da Saúde, Pontifícia Universidade Católica de São Paulo, Sorocaba, SP, Brasil

**Keywords:** anatomy, cadaver, dissection, hand, median nerve, anastomosis, surgical, upper extremity/anatomy and histology

## Abstract

**Objective** To identify the location of the Riché-Cannieu anastomosis (RCA) in relation to the Cardinal Kaplan Line (KCL) and the Y line.

**Methods** A total of 20 hands of 10 recently-deceased adult male cadavers aged between 27 and 66 years were dissected for the investigation of the relationship of the most distal point of the RCA with the KCL and with the Y line, drawn from the axis of the third metacarpal head, following the longitudinal axis of the hand.

**Results** In 20 limbs, the most distal point of the nerve communication was positioned distally in relation to the KCL. The Y line was positioned on the radial side in relation to the most distal point of the RCA in 14 limbs, and it was positioned on the ulnar side in relation to the Y line in 6 limbs. The crossing between the KCL and the Y line occurred proximal to the RCA in 18 limbs; in 1 hand, it was positioned distal to the intersection between these lines; and in another hand, the KCL was positioned exactly on the RCA.

**Conclusion** Knowledge of these anatomical relationships can prevent damage to nerve branches and thus also prevent paralysis of intrinsic muscles in surgical procedures in the palm of the hand.

## Introduction

Some anatomical references are important in the surgical approach to the anatomical structures of the palm. Certain surface lines on the hand enable the identification of noble structures in deep locations. The Kaplan Cardinal Line (KCL) is defined as “a line drawn from the apex of the interdigital crease between the thumb and index finger towards the ulnar side of the hand, to a point 2 cm distal to the pisiform bone”. 1 2

The Riché-Cannieu anastomosis (RCA) was first described by two French anatomists, Riché 3 and Cannieu, 4 as a neural connection between the deep branch of the ulnar nerve and the thenar motor branch of the median nerve. Axons derived from these two nerves can cross and alternate motor innervation of the intrinsic muscles of the hand. The presence of such nerve connections may pose a risk of iatrogenic injury during surgical procedures and make it difficult to interpret electrophysiological studies in the diagnosis of neuropathies. 5

There are three other types of anomalous neural connections between the median and ulnar nerves in the upper limb: the Martin-Gruber anastomosis (in the forearm, communicating nerve fibers originating from the median nerve that go to the ulnar nerve), 6 the Marinacci anastomosis (called reverse anastomosis of Martin-Gruber) 7 and the Berrettini anastomosis (communication between the common digital nerves of the ulnar and median nerves on the palmar surface of the hand). 8 Multiple aberrant connections between the median and ulnar nerves can occur in different combinations. 5

The objective of the present study is to relate the RCA with the KCL and the Y line, to enable safer surgical access when approaching the structures of the palm.

## Materials and Methods

The present study was approved by the institutional Ethics in Research Committee under CAAE: 70113723.1.0000.5373.

A total of 20 hands were dissected from 10 recently deceased adult male cadavers, aged between 27 and 66 years, available at the Department of Anatomy of Pontifícia Universidade Católica de São Paulo (PUC-SP). The dissected hands showed no lesions, deformities, or scars. Dissections were performed with the aid of a magnifying glass (with a magnification of 2.5x). The dissection technique was initiated by an incision proximal to the wrist crease, in the interval between the flexor carpi radialis and palmaris longus muscles, extending distally into the palm of the hand. The palmar skin, subcutaneous tissue and palmar fascia were removed. The superficial and deep flexor tendons were sectioned 2 cm proximally to the flexor retinaculum and distally reflected. The median nerve was identified proximally to the transverse carpal ligament, and the ligament was sectioned longitudinally on its ulnar side. The median nerve branches were dissected distally. The ulnar nerve was also identified in the wrist, proximal to the Guyon canal, and its deep motor branch was followed distally until its communication with branches of the median nerve. With a microsurgical forceps, the terminal fascicles of these two nerves were dissected on the surface of these muscles, or in the thickness of their muscle mass. The distance from the most distal point of the RCA in relation to the KCL and the distal margin of the transverse carpal ligament was measured in 20 limbs of 10 cadavers. A line (Y line) was drawn through the center of the third metacarpal head towards the carpal tunnel, following the longitudinal axis of the hand. The Y line was related to RCA and KCL. Schematic drawings of the pieces were made, which were systematically photographed.

## Results

**Fig. 1:**
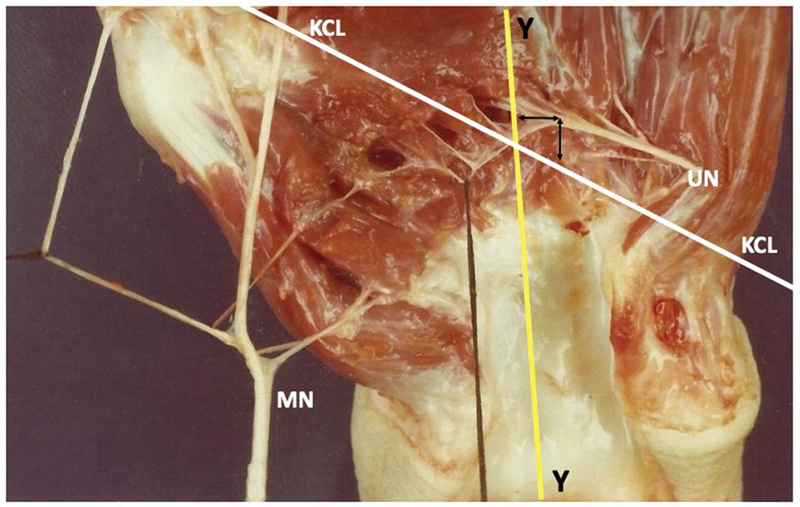
The Y line was positioned on the radial side in relation to the most distal point of the ARC in 14 limbs. In one hand, the intersection between the Kaplan cardinal line (KCL) and the Y line was positioned exactly on the Riché-Cannieu anastomosis (RCA). Abbreviations: MN, median nerve; UN, ulnar nerve.

**Fig. 2:**
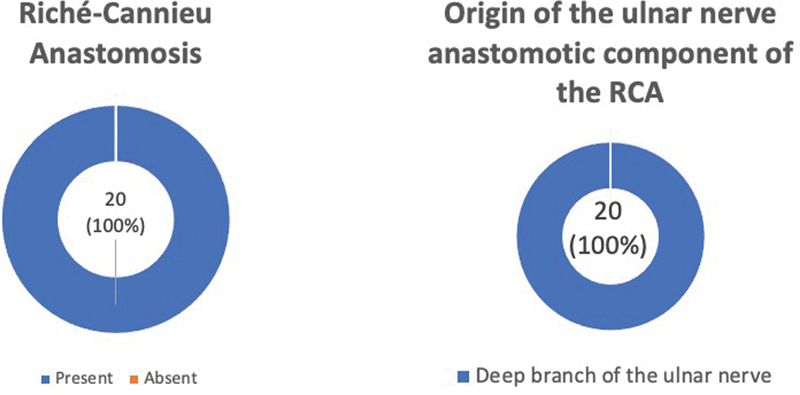
Graphics of the results of the presence of the RCA and origin of the anastomotic component of the ulnar nerve of the RCA.

**Fig. 3:**
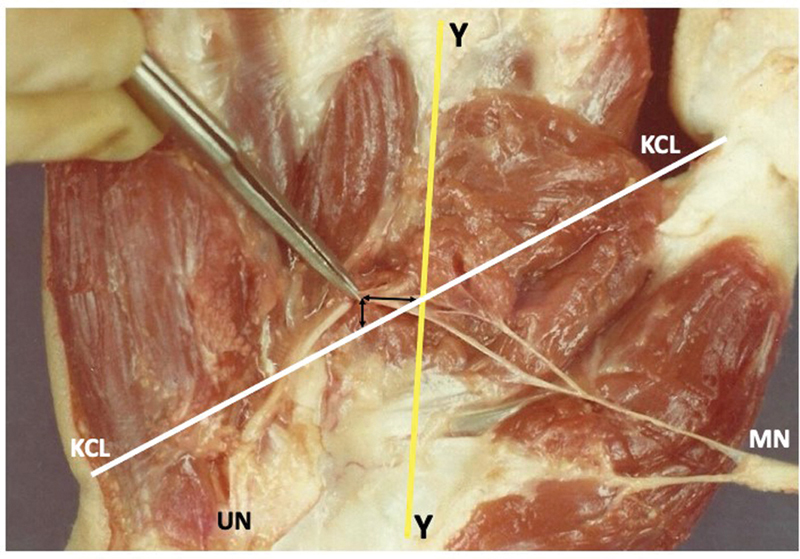
In one hand, the crossing between these lines occurred distal to the RCA. Abbreviations: MN, median nerve; UN, ulnar nerve.

**Fig. 4:**
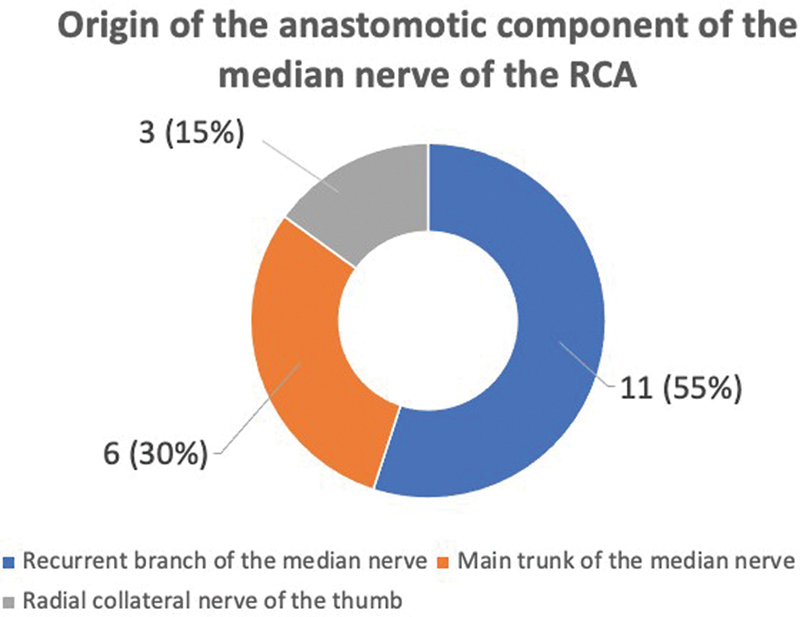
Graph of the results of the origin of the anastomotic component of the median nerve of the RCA.

**Fig. 5:**
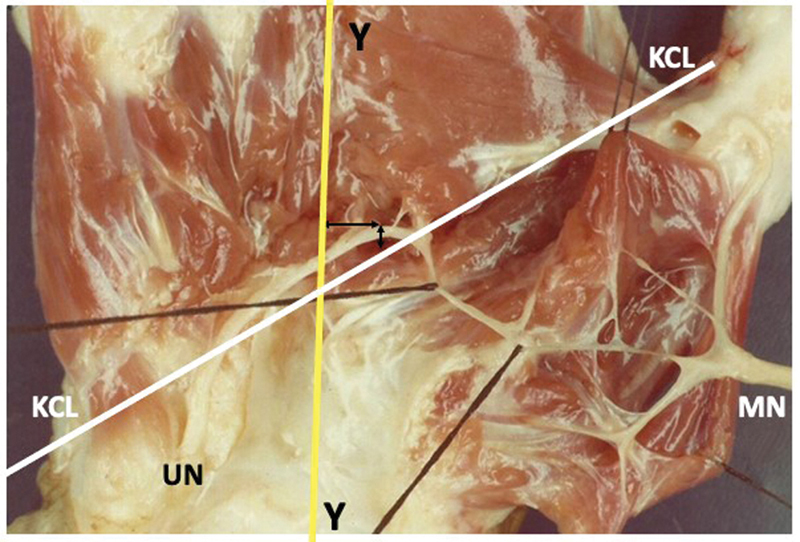
The intersection of the KCL and the Y line was positioned proximal to the RCA. The anastomotic fascicles originated from the main trunk of the median nerve. Abbreviations: MN, median nerve. UN, ulnar nerve.

The RCA was identified in the twenty dissected hands. We observed that the anastomotic branch of the ulnar nerve always came from its deep branch ( Figs. 1 and 2 ). The anastomotic component of the median nerve was represented by fascicles from the recurrent branch of the median nerve in eleven hands ( Figs. 3 and 4 ). In six hands, the anastomotic fascicles originated from the main trunk of the median nerve ( Figs. 4 and 5 ). In three hands, the anastomotic branch originated from the radial collateral nerve of the thumb ( Figs. 1 and 4 ).

**Fig. 6:**
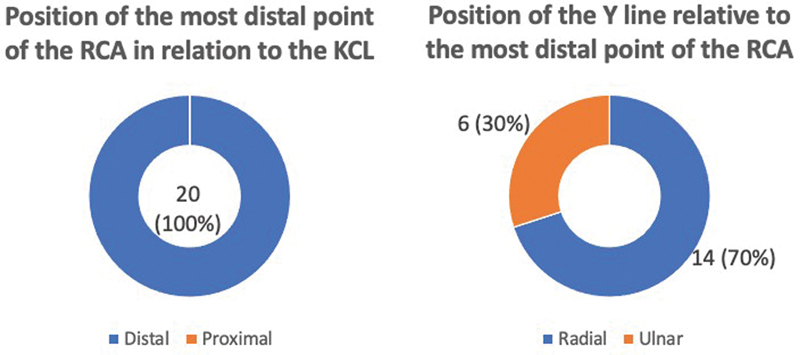
Graphs of the results of the position of the most distal point of the RCA in relation to the KCL and the position of the Y line in relation to the most distal point of the RCA.

**Fig. 7:**
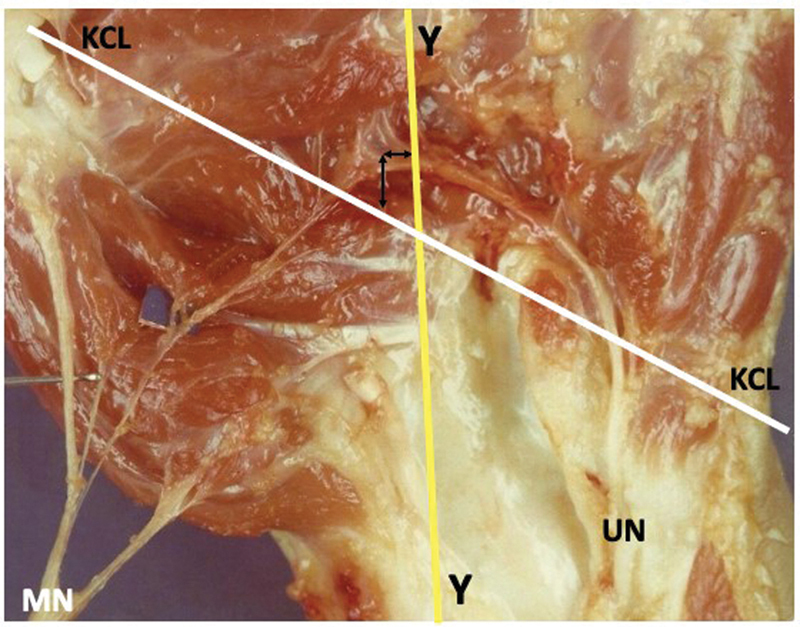
In six hands, the Y line was positioned on the ulnar side in relation to the most distal point of the RCA. Abbreviations: MN, median nerve; UN, ulnar nerve.

**Fig. 8:**
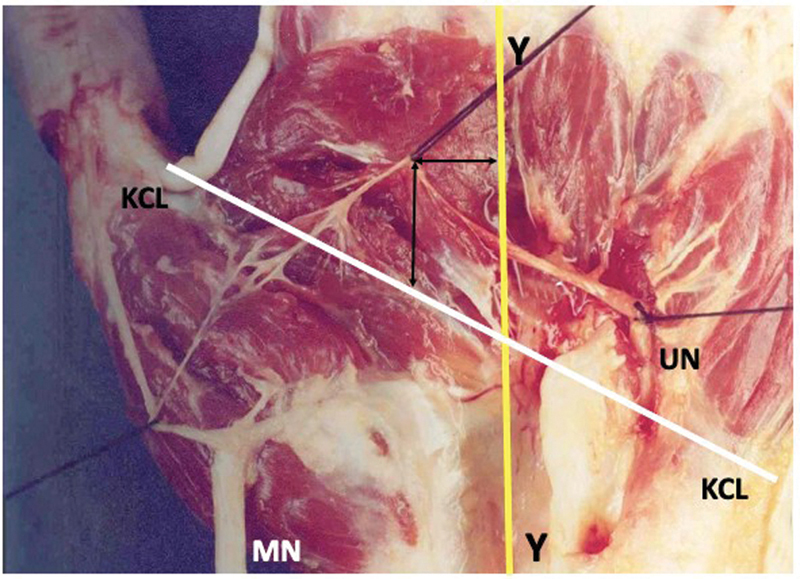
The crossing between the KCL and the Y line occurred proximal to the RCA in 18 limbs. Abbreviations: MN, median nerve.; UN, ulnar nerve.

**Fig. 9:**
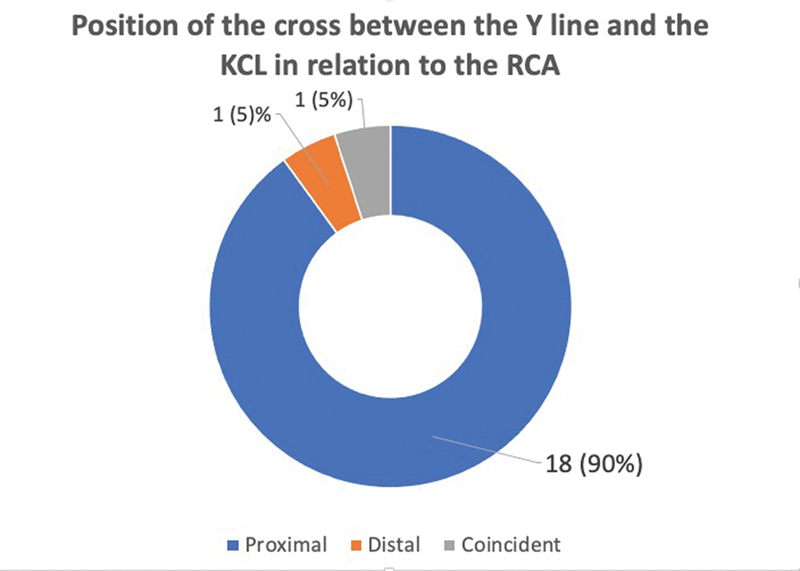
Graph of the results of the position of the crossing between the Y line and the KCL in relation to the RCA.

The most distal point of the RCA was positioned distal in relation to the KCL in the 20 dissected limbs, with a distance ranging from 0.3 cm to 2.5 cm, with a mean of 1.4 cm ( Fig. 6 ). The distance from the RCA to the distal margin of the transverse carpal ligament ranged from 1.3 cm to 3.4 cm, with a mean of 2.4 cm. The Y line was positioned on the radial side in relation to the most distal point of the RCA in 14 limbs, with a distance ranging from 0.2 cm to 1.2 cm, and a mean of 0.5 cm ( Figs. 1 and 6 ). In 6 hands, the Y line was positioned on the ulnar side in relation to the most distal point of the RCA, with a distance ranging from 0.2 mm to 0.8 mm, and a mean of 0.6 mm ( Figs. 6 and 7 ). The crossing between the KCL and the Y line occurred proximal to the RCA in 18 limbs, with a distance of 0.4 cm to 2.7 cm, and a mean of 2.0 cm ( Figs. 8 and 9 ). In 1 hand, the crossing between these lines occurred distal to the RCA, with a distance of 0.4 cm ( Figs. 3 and 9 ). In another hand, the intersection between the KCL and the Y line was positioned exactly on the RCA ( Figs. 1 and 9 ).

## Discussion

Analyzing the literature, we observed that there is no consensus regarding the definition of the KCL, and four different descriptions were found. 1 2 9 10 Vella et al. 9 showed that most surgeons used the KCL as a reference in the surgical procedure. The KCL described by Kaplan 2 in 1966 was considered, and it was drawn from the junction of the line that starts at the apex of the interdigital crease between the thumb and index finger, heading towards the ulnar edge of the hand, up to a point 2 cm distal to the pisiform bone.

In the present study, we identified the RCA in all dissected hands. The most distal point of nerve communication was located distally in relation to the KCL in all limbs. The distance from the RCA to the distal margin of the transverse carpal ligament ranged from 1.3 cm to 3.4 cm, with a mean of 2.4 cm. The Y line drawn from the third metacarpal axis, following the longitudinal axis of the hand, was positioned on the radial side in relation to the most distal point of the RCA in most limbs. The crossing between KCL and the Y line occurred proximal to the RCA in 18 limbs. In one hand, the crossing between the KCL and the Y line occurred distal to the anastomosis, and in another, it was positioned exactly over the RCA.

Analyzing the literature, we identified studies that relate neurovascular structures located in the palm of the hand with the KCL, but we did not identify studies that report the relationship between the RCA and the KCL. Other investigators have used the KCL to describe the location of surgical incisions for procedures such as open carpal tunnel release, 10 11 12 endoscopic carpal tunnel release, 13 and fasciectomy in the treatment of Dupuytren disease. 14

Jurbala and Burbank 15 performed a distal-to-proximal approach to the carpal tunnel using high-resolution ultrasound guidance. The median nerve was visualized longitudinally by ultrasonography within the carpal tunnel; then, the transducer was moved in an ulnar direction in relation to the KCL. The authors 15 reported that this method offers an alternative to carpal tunnel infiltrations. The use of ultrasound guidance enables the surgeon to visualize and prevent damage to neurovascular structures near the median nerve and guide the needle to the site of maximum nerve compression.

Eskandari et al. 16 carried out a study on 37 hands of 34 patients who underwent the carpal tunnel release procedure. A radiological labeling technique was used to determine the location of the thenar motor branch of the median nerve in relation to the KCL.

Ruch et al. 17 related the KCL to the palmar cutaneous nerves of the median and ulnar nerves and reported that a longitudinal incision following the ulnar margin of the middle finger, 2 cm proximal to the KCL, should result in less damage to these nerve branches, and thus reduce the incidence of painful neuromas during open carpal tunnel release.

Dashe and Jones 18 presented a method of exposure and safe removal of the hamate hook in cases of pseudarthrosis with pain symptoms. They 18 used as a reference for the access route to the KCL and the line that accompanies the ulnar margin of the ring finger, to avoid damage to the deep branch of the ulnar nerve.

Some authors have related the KCL with the arterial arches of the palmar surface of the hand. 19 20 21 22 Panchal and Trzeciak 19 performed an anatomical study on 30 cadavers, dissecting 60 hands, to describe the relationship between the KCL and the superficial palmar arterial arch. They believe that, from a clinical point of view, the KCL is the most predictable marker to identify the superficial palmar arch.

McLean et al. 20 carried out an anatomical study on 48 cadaveric hands in specimens aged between 50 and 75 years, with the aim of evaluating the distance between the superficial palmar arch and the KCL. Likewise, Panchal and Trzeciak 19 anatomically correlated the KCL with the superficial and deep palmar arterial arches.

Kwiatkowska et al. 21 dissected twenty upper limbs from cadavers. They related the deep structures of the palm with the creases of the palm of the hand but considered that the palmar creases are very variable between people, and genetics has a lot of influence on the formation of the creases. They consider that the middle palmar crease is parallel to the KCL. Gelberman and North 23 described a more limited incision relating the KCL to the palmar cutaneous branch of the median nerve, considering it to be the ideal access for open carpal tunnel incisions.

In Brazil, we found only one study 24 that related structures of the palm of the hand with the existing creases, in which the authors relate the superficial and deep arterial arches with the wrist creases.

Ma et al. 25 modified the Agee endoscopic portal technique to approach and release the carpal tunnel using the KCL as a reference, and they recorded that the mean distance between the distal margin of the transverse carpal ligament and the KCL ranged from 8.6 mm to 10.7 mm, with an average of 10.0 mm.

## Conclusion

The most distal point of the RCA was positioned distally to the KCL in 20 limbs and, in 14 of them, radially in relation to the Y line. The crossing between the KCL and the Y line occurred proximal to the RCA in 18 limbs. Knowledge of these anatomical relationships can prevent damage to nerve branches and thus also prevent paralysis of intrinsic muscles in surgical procedures in the palm of the hand.
