# Supplementary material for: Anatomical Study of the Relationship between the Riché-Cannieu Anastomosis and the Kaplan Cardinal Line
Source: Rev Bras Ortop (Sao Paulo). 2024 Jun 22;59(3):e409–14. doi: 10.1055/s-0044-1785512 (PMC11193595; doi:10.1055/s-0044-1785512)
Supplement: Related texts

# Research article: Estudo anatômico da relação entre a anastomose de Riché-Cannieu e a linha cardinal de Kaplan

**Keywords:** anastomose cirúrgica, anatomia, cadáver, dissecção, extremidade superior/anatomia e histologia, mão, nervo mediano

## Abstract

**Objetivo** Identificar a localização da anastomose de Riché-Cannieu (ARC) em relação à linha cardinal de Kaplan (LCK) e à linha Y.

**Métodos** Ao todo, 20 mãos de 10 cadáveres adultos do sexo masculino com idades entre 27 e 66 anos, recentemente falecidos, foram dissecadas para a investigação da relação do ponto mais distal da ARC com a LCK e a linha Y traçada a partir do eixo da cabeça do terceiro metacarpo seguindo o eixo longitudinal da mão.

**Resultados** Nos 20 membros, o ponto mais distal da comunicação nervosa era distal à LCK. A linha Y estava no lado radial em relação ao ponto mais distal da ARC em 14 membros, e no lado ulnar em relação à linha Y em 6 membros. O cruzamento entre a LCK e a linha Y foi proximal à ARC em 18 membros; em 1 mão, era distal à intersecção entre essas linhas e, em outra mão, a LCK estava exatamente na ARC.

**Conclusão** O conhecimento dessas relações anatômicas pode prevenir danos aos ramos nervosos e, assim, também prevenir a paralisia dos músculos intrínsecos em procedimentos cirúrgicos na palma da mão.

## Introdução

Algumas referências anatômicas são importantes na abordagem cirúrgica das estruturas anatômicas da palma. Certas linhas superficiais da mão permitem a identificação de estruturas nobres de localização profunda. A linha cardinal de Kaplan (LCK) é definida como “uma linha traçada do ápice da prega interdigital entre o polegar e o dedo indicador em direção ao lado ulnar da mão, até um ponto 2 cm distal ao osso pisiforme”. 1 2

A anastomose de Riché-Cannieu (ARC) foi descrita pela primeira vez por dois anatomistas franceses, Riché 3 e Cannieu, 4 como uma conexão neural entre o ramo profundo do nervo ulnar e o ramo motor tenar do nervo mediano. Os axônios derivados desses dois nervos podem se cruzar e alternar a inervação motora dos músculos intrínsecos da mão. A presença de tais conexões nervosas podem representar risco de lesão iatrogênica durante procedimentos cirúrgicos e dificultar a interpretação de estudos eletrofisiológicos no diagnóstico de neuropatias. 5

Existem três outros tipos de conexões neurais anômalas entre os nervos mediano e ulnar no membro superior: a anastomose de Martin-Gruber (no antebraço, em que fibras nervosas comunicantes originadas do nervo mediano vão para o nervo ulnar), 6 a anastomose de Marinacci (chamada de anastomose reversa de Martin-Gruber) 7 e a anastomose de Berrettini (comunicação entre os nervos digitais comuns dos nervos ulnar e mediano na superfície palmar da mão). 8 Múltiplas conexões aberrantes entre os nervos mediano e ulnar podem ocorrer em diferentes combinações. 5

O objetivo deste estudo é relacionar a ARC com a LCK e a linha Y, para permitir o acesso cirúrgico com mais segurança na abordagem das estruturas da palma.

## Materiais e Métodos

O presente estudo foi aprovado pelo Comitê de Ética em Pesquisa institucional sob número CAAE 70113723.1.0000.5373.

Ao todo, 20 mãos de 10 cadáveres adultos do sexo masculino, recentemente falecidos, com idade entre 27 e 66 anos, disponíveis no Departamento de Anatomia da Pontifícia Universidade Católica de São Paulo (PUC-SP), foram dissecadas. As mãos dissecadas não apresentavam lesões, deformidades ou cicatrizes. As dissecções foram realizadas com o auxílio de lupa (com aumento de 2,5x). A técnica de dissecção foi iniciada por uma incisão proximal à prega do punho, no intervalo entre os músculos flexor radial do carpo e palmar longo, que se estendeu distalmente na palma da mão. A pele palmar, o tecido subcutâneo e a fáscia palmar foram removidos. Os tendões flexores superficiais e profundos foram seccionados 2 cm proximalmente ao retináculo flexor e refletidos distalmente. O nervo mediano foi identificado proximalmente ao ligamento transverso do carpo, que foi seccionado em sentido longitudinal em seu lado ulnar. Os ramos do nervo mediano foram dissecados distalmente. O nervo ulnar também foi identificado no punho, proximal ao canal de Guyon, e seu ramo motor profundo foi acompanhado distalmente até a sua comunicação com ramos do nervo mediano. Com uma pinça microcirúrgica, os fascículos terminais desses dois nervos foram dissecados na superfície desses músculos ou na espessura de sua massa muscular. A distância do ponto mais distal da ARC em relação à LCK e à margem distal do ligamento transverso do carpo foi medida nos 20 membros dos 10 cadáveres. Uma linha (linha Y) foi traçada do centro da cabeça do terceiro metacarpo em direção ao túnel do carpo, seguindo o eixo longitudinal da mão. A linha Y estava relacionada à ARC e à LCK. Desenhos esquemáticos das peças foram preparados e sistematicamente fotografados.

## Resultados

A ARC foi identificada em todas as vinte mãos dissecadas. Observamos que o ramo anastomótico do nervo ulnar sempre se originava do seu ramo profundo ( Figs. 1 e 2 ). O componente anastomótico do nervo mediano era representado por fascículos do ramo recorrente do nervo mediano em onze mãos ( Figs. 3 e 4 ). Em seis mãos, os fascículos anastomóticos originavam-se do tronco principal do nervo mediano ( Figs. 4 e 5 ). Em três mãos, o ramo anastomótico se originava do nervo colateral radial do polegar ( Figs. 1 e 4 ).

**Fig. 1:** A linha Y estava do lado radial em relação ao ponto mais distal do arco em 14 membros. Em uma mão, a intersecção entre a linha cardinal de Kaplan (LCK) e a linha Y estava exatamente na anastomose de Riché-Cannieu (ARC). Abreviaturas: MN, nervo mediano; UN, nervo ulnar.

**Fig. 2:** Gráficos dos resultados da presença da ARC e origem do componente anastomótico do nervo ulnar da ARC.

**Fig. 3:** Em uma mão, o cruzamento entre essas linhas ocorreu distalmente à ARC. Abreviaturas: MN, nervo mediano; UN, nervo ulnar.

**Fig. 4:** Gráfico dos resultados da origem do componente anastomótico do nervo mediano da ARC.

**Fig. 5:** A intersecção entre a LCK e a linha Y era proximal à ARC. Os fascículos anastomóticos originaram-se do tronco principal do nervo mediano. Abreviaturas: MN, nervo mediano; UN, nervo ulnar.

O ponto mais distal da ARC era distal em relação à LCK nos 20 membros dissecados, com distância entre 0,3 cm e 2,5 cm e média de 1,4 cm ( Fig. 6 ). A distância da ARC à margem distal do ligamento transverso do carpo variou de 1,3 cm a 3,4 cm, com média de 2,4 cm. A linha Y estava do lado radial em relação ao ponto mais distal da ARC em 14 membros, com distância entre 0,2 cm e 1,2 cm e média de 0,5 cm ( Figs. 1 e 6 ). Em 6 mãos, a linha Y estava no lado ulnar em relação ao ponto mais distal da ARC, com distância entre 0,2 mm e 0,8 mm, com média de 0,6 mm ( Figs. 6 e 7 ). O cruzamento entre a LCK e a linha Y ocorreu proximal à ARC em 18 membros, com distância de 0,4 cm a 2,7 cm e média de 2,0 cm ( Figs. 8 e 9 ). Em 1 mão, o cruzamento entre essas linhas ocorreu distalmente à ARC, com distância de 0,4 cm ( Figs. 3 e 9 ). Em outra mão, a intersecção entre a LCK e a linha Y estava exatamente na ARC ( Figs. 1 e 9 ).

**Fig. 6:** Gráficos dos resultados da posição do ponto mais distal da ARC em relação à LCK e da posição da linha Y em relação ao ponto mais distal da ARC.

**Fig. 7:** Em seis mãos, a linha Y estava do lado ulnar em relação ao ponto mais distal da ARC. Abreviaturas: MN, nervo mediano; UN, nervo ulnar.

**Fig. 8:** O cruzamento entre a LCK e a linha Y era proximal à ARC em 18 membros. Abreviaturas: MN, nervo mediano; UN, nervo ulnar.

**Fig. 9:** Gráfico dos resultados da posição do cruzamento entre a linha Y e a LCK em relação à ARC.

## Discussão

Analisando a literatura, observamos que não há consenso quanto à definição da LCK, pois encontramos quatro descrições diferentes. 1 2 9 10 Vella et al. 9 mostraram que a maioria dos cirurgiões utilizou a LCK como referência no procedimento cirúrgico. A LCK considerada foi a descrita por Kaplan 2 em 1966, sendo traçada a partir da junção da linha que começa no ápice da prega interdigital entre o polegar e o indicador e seguindo em direção à borda ulnar da mão, até o ponto 2 cm distal ao osso pisiforme. 2

Neste estudo, identificamos a ARC em todas as mãos dissecadas. O ponto mais distal de comunicação nervosa era distal à LCK em todos os membros. A distância entre a ARC e a margem distal do ligamento transverso do carpo variou de 1,3 cm a 3,4 cm, com média de 2,4 cm. A linha Y traçada a partir do terceiro eixo metacárpico, seguindo o eixo longitudinal da mão, estava no lado radial em relação ao ponto mais distal da ARC na maioria dos membros. O cruzamento entre a LCK e a linha Y ocorreu proximal à ARC em 18 membros. Em uma mão, o cruzamento entre a LCK e a linha Y era distal à anastomose e, em outra, estava exatamente sobre a ARC.

Analisando a literatura, identificamos estudos que relacionam estruturas neurovasculares localizadas na palma da mão com a LCK, mas não encontramos estudos sobre a relação entre a ARC e a LCK. Outros investigadores usaram a LCK para descrever a localização de incisões cirúrgicas para procedimentos como liberação aberta do túnel do carpo, 10 11 12 liberação endoscópica do túnel do carpo 13 e fasciectomia para tratamento da doença de Dupuytren. 14

Jurbala e Burbank 15 realizaram uma abordagem distal-proximal ao túnel do carpo utilizando orientação ultrassonográfica de alta resolução. O nervo mediano foi visualizado longitudinalmente por ultrassonografia dentro do túnel do carpo, e, em seguida, o transdutor foi movido na direção ulnar com relação à LCK. Esses autores 15 relataram que esse método é uma alternativa às infiltrações do túnel do carpo. A orientação ultrassonográfica permite ao cirurgião visualizar e prevenir danos às estruturas neurovasculares próximas ao nervo mediano e guiar a agulha até o sítio de compressão máxima do nervo.

Eskandari et al. 16 estudaram 37 mãos de 34 pacientes submetidos ao procedimento de liberação do túnel do carpo. Uma técnica de marcação radiológica determinou a localização do ramo motor tenar do nervo mediano em relação à LCK.

Ruch et al. 17 relacionaram a LCK aos nervos cutâneos palmares dos nervos mediano e ulnar, e relataram que uma incisão longitudinal seguindo a margem ulnar do dedo médio, 2 cm proximal à LCK, deveria gerar menor dano a esses ramos nervosos e, assim, reduzir a incidência de neuromas dolorosos durante a liberação aberta do túnel do carpo.

Dashe e Jones 18 apresentaram um método de exposição e remoção segura do hâmulo do hamato em casos de pseudartrose com sintomas álgicos. Esses autores 18 utilizaram como referência a via de acesso à LCK e a linha que acompanha a margem ulnar do dedo anular para evitar lesão do ramo profundo do nervo ulnar.

Alguns autores 19 20 21 22 relacionaram a LCK aos arcos arteriais da superfície palmar da mão. Panchal e Trzeciak 19 realizaram um estudo anatômico em 30 cadáveres, e dissecaram 60 mãos para descrever a relação entre a LCK e o arco arterial palmar superficial. Esses autores 19 acreditam que, do ponto de vista clínico, a LCK é o marcador mais previsível para a identificação do arco palmar superficial.

McLean et al. 20 realizaram estudo anatômico em 48 mãos de cadáveres entre 50 e 75 anos de idade com o objetivo de avaliar a distância entre o arco palmar superficial e a LCK. Da mesma forma, Panchal e Trzeciak 19 correlacionaram anatomicamente a LCK aos arcos arteriais palmares superficiais e profundos

Kwiatkowska et al. 21 dissecaram vinte membros superiores de cadáveres, e relacionaram as estruturas profundas da palma com as pregas da palma da mão, mas consideraram que as pregas palmares são muito variáveis entre os indivíduos, e que a genética tem muita influência em sua formação. Eles consideram que a prega palmar média é paralela à LCK. Gelberman e North 23 descreveram uma incisão mais limitada, relacionando a LCK ao ramo cutâneo palmar do nervo mediano, considerando-a o acesso ideal para incisões abertas no túnel do carpo.

No Brasil, encontramos apenas um estudo 24 que relacionou estruturas da palma da mão com as pregas existentes; nesse estudo, os autores relacionaram os arcos arteriais superficiais e profundos com as pregas do punho.

Ma et al. 25 modificaram a técnica de portal endoscópico de Agee para a abordagem e liberação do túnel do carpo usando a LCK como referência, e registraram que a distância média entre a margem distal do ligamento transverso do carpo e a LCK variou de 8,6 mm a 10,7 mm, com média de 10,0 mm.

## Conclusão

O ponto mais distal da ARC era distal à LCK em 20 membros e, em 14 deles, era radial em relação à linha Y. O cruzamento entre a LCK e a linha Y era proximal à ARC em 18 membros. O conhecimento dessas relações anatômicas pode prevenir danos aos ramos nervosos e, assim, também prevenir a paralisia dos músculos intrínsecos em procedimentos cirúrgicos na palma da mão.
